# A turn-on endoplasmic reticulum-targeted two-photon fluorescent probe for hydrogen sulfide and bio-imaging applications in living cells, tissues, and zebrafish

**DOI:** 10.1038/s41598-017-13325-z

**Published:** 2017-10-11

**Authors:** Yonghe Tang, An Xu, Yanyan Ma, Gaoping Xu, Shiying Gao, Weiying Lin

**Affiliations:** Institute of Fluorescent Probes for Biological Imaging, School of Chemistry and Chemical Engineering, School of Materials Science and Engineering, University of Jinan, Jinan, Shandong 250022 P.R. China

## Abstract

As one of the important gas signal molecules, hydrogen sulfide (H_2_S) is associated with many important physiological processes in living organisms. Organelles, especially endoplasmic reticulum (ER), play a crucial role in the cell metabolism. Accordingly, the detection of H_2_S in the ER is of high interest. Toward this goal, we have described the development of the first ER-targeted fluorescent H_2_S probe (**Na-H**_**2**_ **S-ER**). The new probe exhibited favorable features, such as a large turn-on fluorescence signal (45-fold fluorescence enhancement), high sensitivity and selectivity. The probe was successfully employed for imaging exogenous and endogenous H_2_S in the living HeLa cells. Significantly, the new probe **Na-H**_**2**_ **S-ER** was employed to visualize H_2_S in the ER of living cells for the first time. In addition, the probe was also successfully used for imaging H_2_S in the living tissues up to a depth of 100 μm and in the living zebrafish.

## Introduction

Although hydrogen sulfide (H_2_S) is an important industrial raw material in the sulfide industry, it is also a serious hazardous substance in the industrial process. For example, H_2_S could seriously corrode metallic equipment^1^. Early researches suggest that H_2_S has been regarded as a poisonous and harmful gas^2^. Prolonged exposure to H_2_S may lead to a series of malignant diseases, such as Down syndrome^3^, Alzheimer’s disease^4^, diabetes^5^, and liver cirrhosis^6^. However, recent studies show that H_2_S is also a gas signal molecule and plays the important physiological functions as the same as nitric oxide (NO) and carbon monoxide (CO) in organisms^7,8^. Endogenous H_2_S is involved in many physiological activities including regulating cardiovascular systems, influencing the proliferation and apoptosis of human cells, stabilizing the nervous and the immune system. Medical researches show that endogenous H_2_S is produced from L-cysteine by the enzyme-catalytic reaction of cystathionine-*γ*-lyase (CSE), cystathionine-*β*-synthetase (CBS), and 3-mercaptopyruvate sulfurtransferase (3-MST)^9–11^. Although H_2_S is associated with different physiological processes, most of the physiological functions of H_2_S are still unknown. Therefore, the development of detection methods for H_2_S with high sensitivity and good selectivity is very valuable in complicated biological systems.

Nowadays, there are some analytical methods for H_2_S including colorimetry^12^, electrochemical assay^13^, gas chromatography^14^, and sulfide precipitation^15^. However, these techniques can’t realize the instantaneous monitoring of H_2_S. In addition, the tissues or cells samples need to be destroyed in the detection process. By comparison, the fluorescence imaging methods have many advantages, such as high selectivity and sensitivity, and undamaged samples in the detection process^16–19^. In fluorescence imaging, the two-photon fluorescence imaging has drawn much attention because of low photo-damage sample, deep penetration, and low background fluorescence^20–22^.

In the eukaryotic cells, the endoplasmic reticulum (ER) is the largest organelle with single-membrane structure^23^. The ER performs a variety of functions in living cells, including synthesizing, processing, and modifying protein and lipid, stabilizing intracellular Ca^2+^ concentrations^24^. Biologist studies show that the ER is closely associated with other organelles, including golgi apparatus, cell membrane, and mitochondria^25–27^. So far, many fluorescent probes have been reported for H_2_S detection in the past decade^28–39^. However, because of lack of the ER-specific fluorescent probes, the detection of physiological function of H_2_S in the ER is still very challenging.

Herein, we report the first ER-targeted H_2_S fluorescent probe **Na-H**_**2**_ **S-ER**. The probe contains the fluorescent platform 1,8-naphthalimide, and the H_2_S recognition site azide group. Combined with the methyl sulfonamide group, the fluorescent probe **Na-H**_**2**_ **S-ER** preferentially accumulated in the ER^40–43^. Through the spectrum analysis, the probe **Na-H**_**2**_ **S-ER** shows excellent selectivity and sensitivity to H_2_S relative to other analytes, and the probe can be used to detect H_2_S in cells. In addition, the probe **Na-H**_**2**_ **S-ER** is suitable for fluorescence imaging of H_2_S in living tissues and zebrafish.

## Results and Discussion

### Design and synthesis

**Figure 1:**
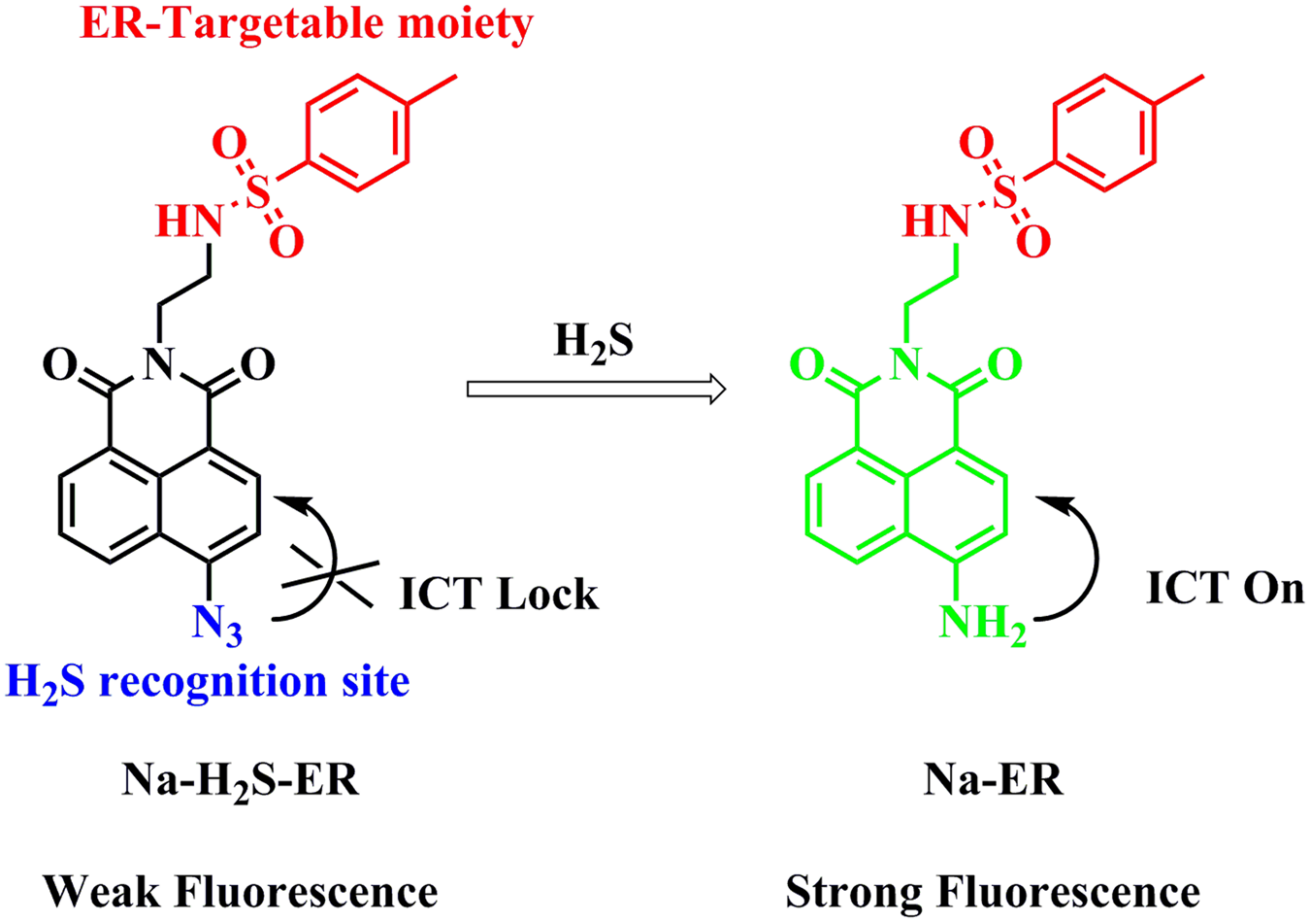
The structure of the probe **Na-H**_**2**_ **S-ER** and the proposed recognition mechanism for H_2_S.

Naphthylamide dye is a classical fluorescence platform and has excellent two-photon properties. We used naphthylamide and introduced the H_2_S identification site azide group to construct a fluorescent H_2_S probe, named as **Na-H**_**2**_ **S-ER**. Attach the strong electron-withdrawing azide group to the naphthalimide fluorescent platform directly, the electronic structure of the probe was shown as “A-π-A”. This electronic structure made the fluorescent probe have a weak fluorescent emission signal. When the probe was treated with H_2_S, the azide group became an amine moiety, and the electronic structure of the probe become “D-π-A” because of the amine moiety was the strong electron-donating group. Due to the intramolecular charge transfer (ICT) effect, the fluorescent probe had strong fluorescent emission (Fig. 1). Sulfonylurea compounds are inhibitors of ATP-sensitive K^+^ channels, and thus can selectively bind to these proteins that are prominent on ER. Therefore, fluorescent analogues of sulfonylurea are usually used to label ER. As shown in Fig. S1, glyburide was used as the targeting group in ER-tracker Green and ER-tracker Red. In contrast, in ER-tracker Blue, sulfanilamide, an analogue of sulfonylurea, was used as the recognition group. Therefore, in this work, we have also used sulfanilamide as the targeting group, and constructed the probe that can label ER due to selectively binding to ATP-sensitive K^+^ channels. The synthetic route of the fluorescent probe was shown in Fig. S2. The new compound **Na-H**_**2**_ **S-ER** was fully characterized by ^1^H NMR, ^13^C NMR and HR-MS.

### Optical properties of probe Na-H_2_S-ER

**Figure 2:**
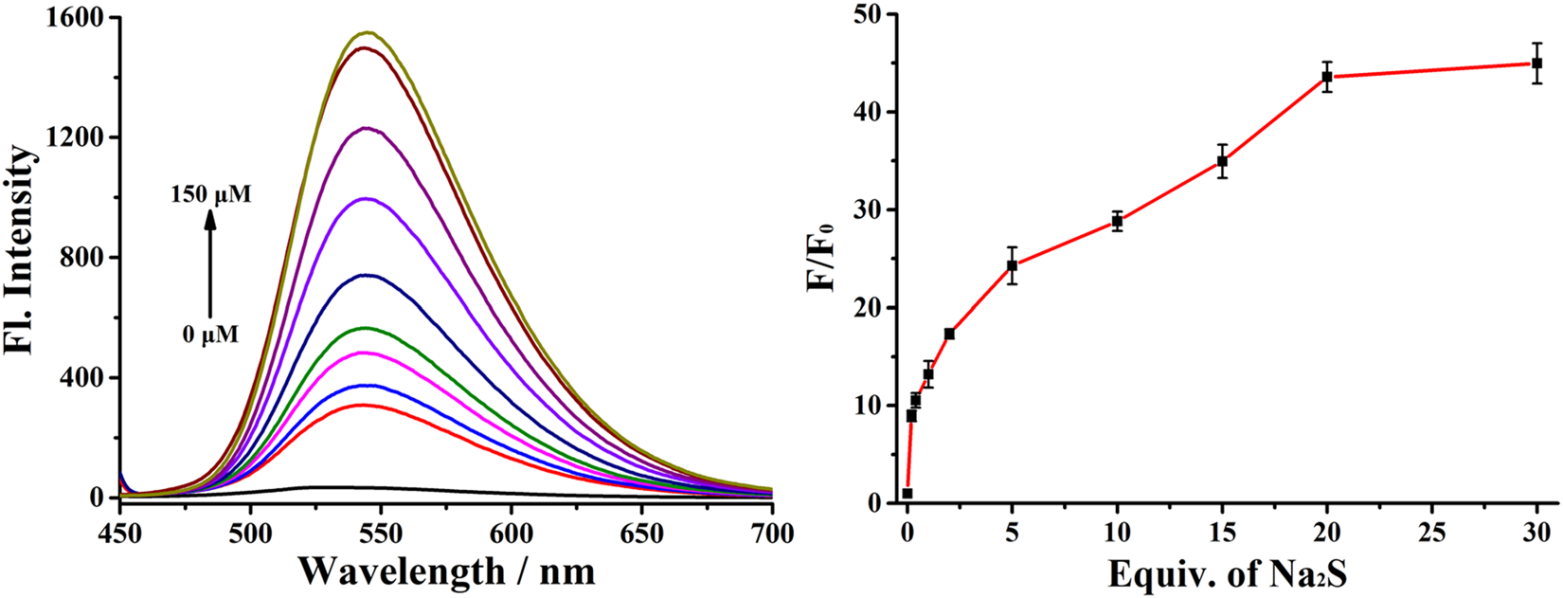
Fluorescence spectra of the probe Na-H_2_S-ER (5 μM) in 10 mM PBS buffer (pH 7.4, 5**%** DMSO) with various concentrations of Na_2_S.

After synthesizing the probe, we first tested its absorption spectra with different concentration of Na_2_S (acknowledged as a release reagent of H_2_S) in PBS buffer (pH 7.4, 5% DMSO). The maximum absorption wavelength of the probe **Na-H**_**2**_ **S-ER** was at about 385 nm (ε = 9,200 M^−1^ cm^−1^) (Fig. S3). With the increase of the introduction of the concentration of Na_2_S (0–20 equiv.), the absorption value of the probe at 385 nm gradually reduced, and the absorption value at 440 nm gradually increased. Similar to the absorption spectrum, the fluorescence emission spectrum of the probe **Na-H**_**2**_ **S-ER** also changed significantly. As designed, the free probe was almost non-fluorescent because of the specific electronic structure of the probe. However, when titrated with Na_2_S, a significant fluorescence turn-on response at 545 nm was observed (Fig. 2). The large fluorescence enhancement (up to 45-fold) was observed when the probe was treated with Na_2_S (20 equiv.) for 30 min at room temperature (Fig. 2). Based on the titration experiments, the detection limit of the probe for H_2_S was calculated to be 7.77 × 10^−6^ M (Fig. S4), indicating that the fluorescent probe has a potential application value for detecting H_2_S due to it could detect the dynamic changes of H_2_S concentrations in the living system.

### Mechanism

The compound **Na-ER** (the reaction product of the probe with Na_2_S) was synthesized to further verify the design strategy of the probe **Na-H**_**2**_ **S-ER**. **Na-ER** was purified by the column chromatography, and the structure was determined by NMR (Figs S5 and S6) analysis and HR-MS spectrometry (Fig. S7). These results confirmed that the recognition mechanism of the probe with H_2_S as proposed we expected (Fig. 1).

### Dynamic research

**Figure 3:**
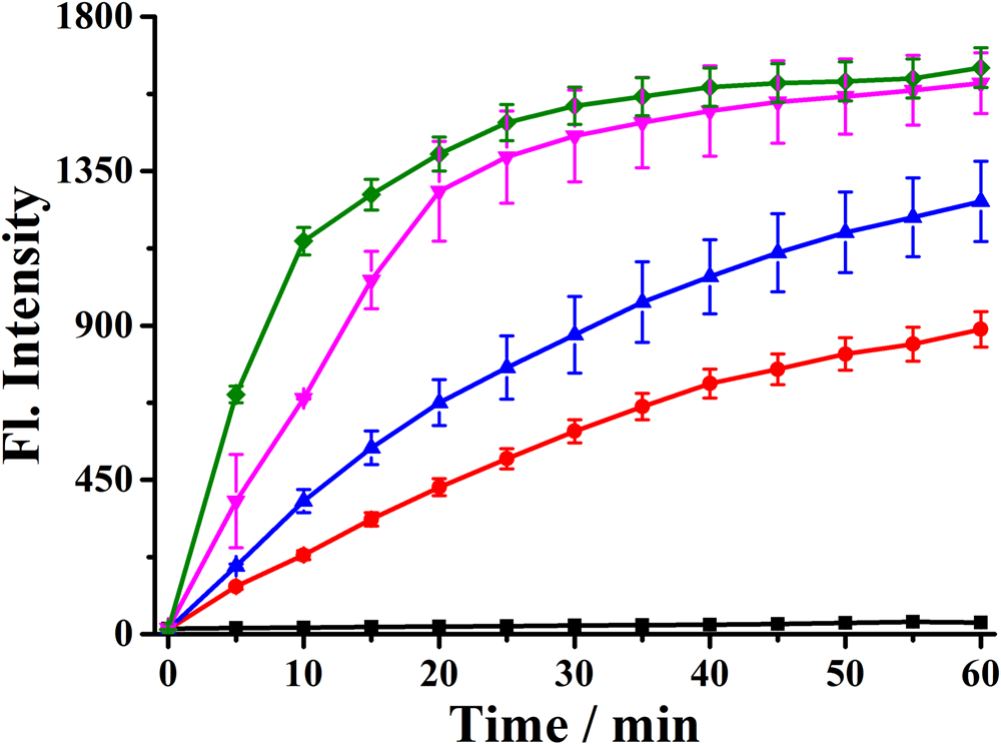
Reaction-time profiles of the probe **Na-H**_2_ **S-ER** (5 μM) in the absence [■] or presence of Na_2_S (10 μM , 25 μM , 100 μM , and 150 μM ). The fluorescence intensities at 545 nm were continuously monitored at time intervals in PBS buffer.

The rate of response is a basic parameter of the probe recognition capability, we then carried out the dynamic test of the probe **Na-H**_**2**_ **S-ER** (5 μM) reacted with various concentrations of Na_2_S (Fig. 3). Untreated with Na_2_S, the fluorescence intensity of the probe had almost no change within 60 min. By contrast, the fluorescence intensity changed significantly when the addition of Na_2_S. The intensity essentially reached a maximum in 30 min at room temperature when the probe **Na-H**_**2**_ **S-ER** (5 μM) reacted with Na_2_S (100 μM). Under the *pseudo*-first-order conditions, the rate constant for the probe was determined to be *k* = 0.0702 min^−1^ (Fig. S8), suggesting that the probe may be used to detect H_2_S for real-time imaging applications in living systems.

### Selectivity study

**Figure 4:**
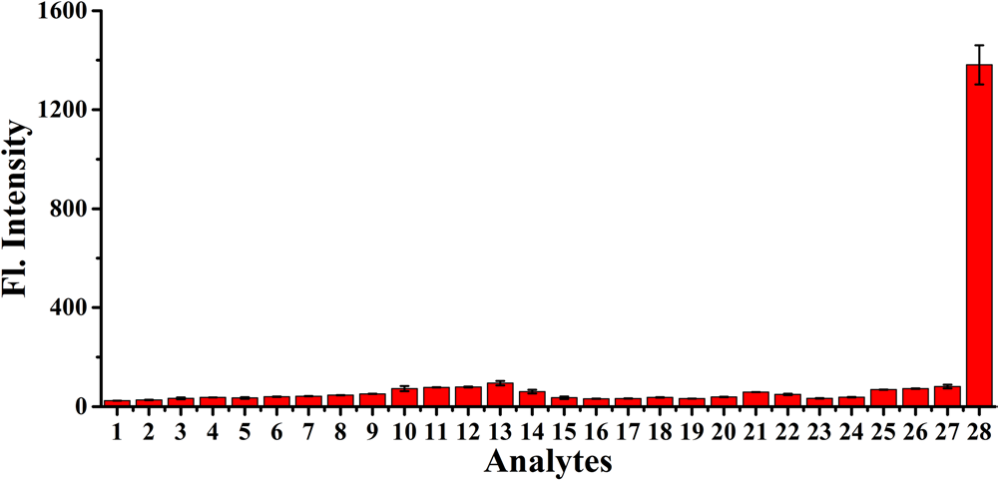
Fluorescence responses of **Na-H**_2_ **S-ER** (5 μM) in the presence of various relevant analytes in 10 mM PBS buffer (pH 7.4, 5**%** DMSO). The concentrations of the representative analytes are: 100 μM. Legend: (1) probe; (2) NaBr; (3) NaNO_2_; (4) MgCl_2_; (5) NaNO_3_; (6) Zn(OAc)_2_; (7) ZnCl_2_; (8) KF; (9) NaHSO_3_; (10) KSCN; (11) KI; (12) Na_2_S_2_O_3_; (13) FeSO_4_; (14) N_2_H_4_; (15) TBHP; (16) DTBP; (17) H_2_O_2_; (18) NaClO; (19) NO; (20) sodium citrate; (21) glucose; (22) VC; (23) Gly; (24) Ala; (25) GSH; (26) Hcy; (27) Cys; (28) Na_2_S. λ_ex_/_em_ = 440/545 nm.

The high selectivity of probe makes it potentially useful for biomedical application. To investigate the selectivity, **Na-H**_**2**_ **S-ER** (5 μM) was treated with various relevant analytes, including various anions/cations, representative amino acid, reactive oxygen/nitrogen species (ROS/RNS) in 10 mM PBS buffer (pH 7.4, 5% DMSO) (Fig. 4). The addition of the anions (Br^−^, NO_2_^−^, Cl^−^, NO_3_^−^, AcO^−^, F^−^, HSO_3_^−^, SCN^−^, I^−^, S_2_O_3_^2−^, SO_4_^2−^) and cations (Na^+^, K^+^, Mg^2+^, Zn^2+^, Fe^2+^), ROS/RNS (H_2_O_2_, NaClO, *tert*-butylhydroperoxide (TBHP), *di-tert*-butylperoxide (DTBP), NO), representative amino acid (glycine (Gly), alanine (Ala), cysteine (Cys), homocysteine (Hcy), glutathione (GSH)), and other analytes (Vitamin C (VC), sodium citrate) at 100 μM caused almost no change of the fluorescence intensity of the probe **Na-H**_**2**_ **S-ER** at room temperature for 30 min. By contrast, upon treatment of Na2S (100 μM) with the probe, the significant change occurred in the fluorescence intensity of the probe (about 45-fold fluorescence enhancement). These results showed that the probe has a high selectivity for H_2_S over the other tested analytes, suggesting that probe had the potential to detect hydrogen sulfide in living biological systems.

### The pH and photostability studies

The pH and photostability are all related to the application of the probe. We then examined the possible effects of different pH on the fluorescence changes of the probe **Na-H**_**2**_ **S-ER** in the absence or presence of Na_2_S (Fig. S9). The fluorescence intensity of the probe was almost unchanged over a wide pH range of 4–10 range in the absence of Na_2_S, indicating that the probe was almost impervious to the effect of pH. However, when Na_2_S was introduced, a remarkable fluorescent signal enhancement was observed at about 545 nm, especially under the physiological conditions (pH 7.4). These results suggested that the probe can detect H_2_S in different pH environments, indicating that the probe has the potential value for biological applications. Furthermore, the photostability experiment was also carried out. After excited by the short wavelength UV light (365 nm), the fluorescent intensity of the probe displayed slight variation (Fig. S10), suggesting that the probe has good photostability and may have potential usefulness in living system.

### Cytotoxicity and bio-imaging H_2_S in living HeLa cells

As a potential imaging agent, the probe **Na-H**_**2**_ **S-ER** should have low toxicity. We carried out a cytotoxicity test for the fluorescence probe using the standard MTT assay (Fig. S11). The results showed that the probe has no remarkable cytotoxicity to the cells in low doses (0–20 μM) after 24 h of incubation, meaning that the probe can be used for imaging experiments.

Because the probe had many excellent properties, including high sensitivity and selectivity, good photostability, low cytotoxicity, and working appropriately at the physiological pH, we would carry out the fluorescence imaging experiments in living cells. Because of the 1,8-naphthalimide is the classic two-photon dye, the fluorescence imaging experiments were carried out by the one-photon (OP) mode and two-photon (TP) mode. There was no fluorescent signal was detected when the HeLa cells were only incubated with probe (5 μM) whether by OP or TP mode (Fig. S12b,d). By contrast, when the HeLa cells were pre-incubated with probe (5 μM), and then treated with Na_2_S (50 μM), a very strong fluorescent signals could be detected by OP and TP excitation (Fig. S12f,h), confirming that the fluorescence probe has good biocompatibility and it is able to recognize the exogenous H_2_S in the living cells.

**Figure 5:**
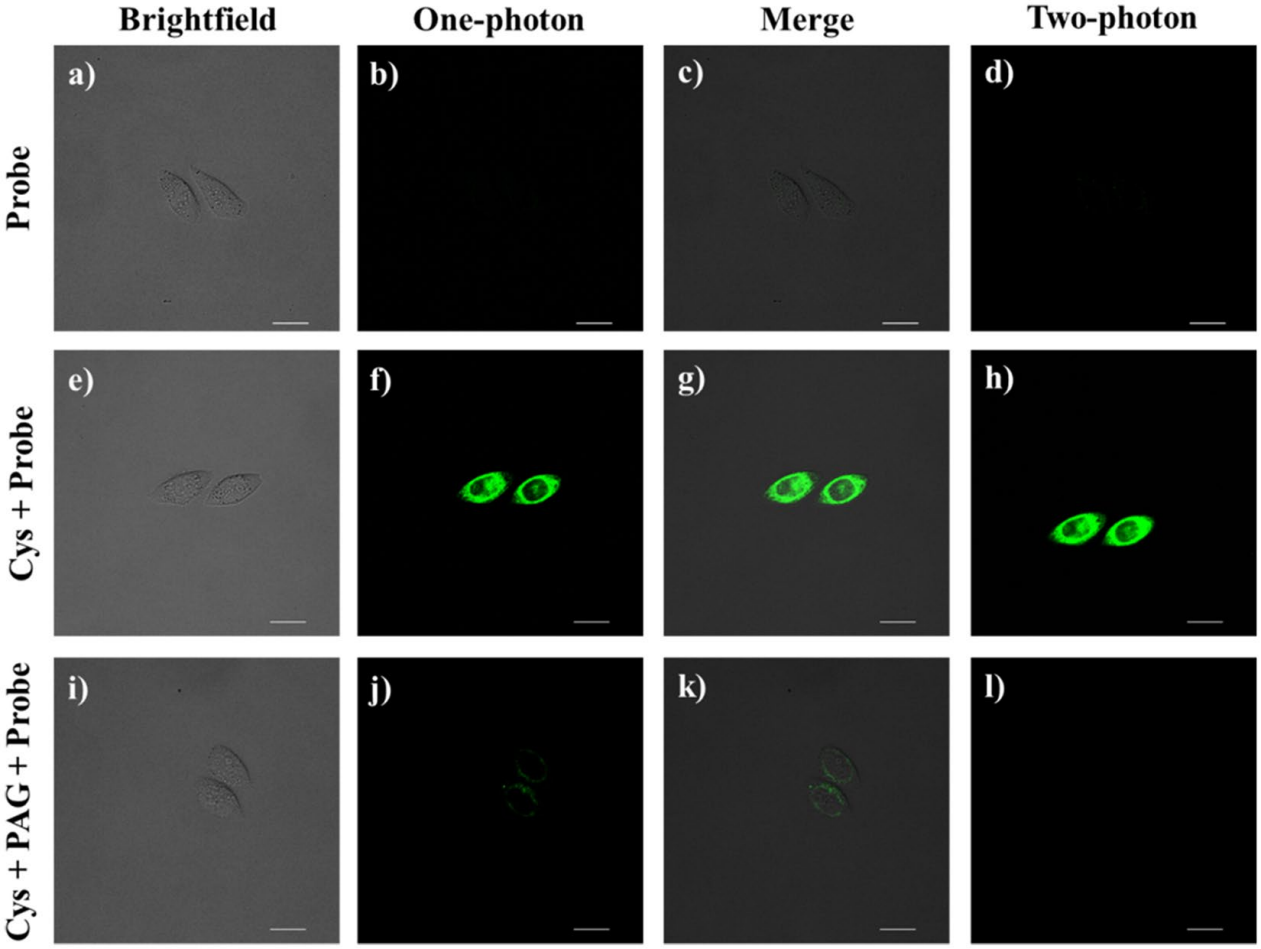
Fluorescence imaging of the endogenous H_2_S in the HeLa cells. (**a**) The brighfield image of the HeLa cells treated with **Na-H**_**2**_ **S-ER** (5 μM); (**b**) The OP Fluorescence image of a; (**c**) The merge image of (**a** and **b–d**) The TP Fluorescence image of a; (**e**) The brighfield image of the HeLa cells treated with Cys (100 μM) and **Na-H**_**2**_ **S-ER** (5 μM); (**f**) The OP Fluorescence image of (**e**,**g**) The merge image of (**e** and **f**,**h**) The TP Fluorescence image of (**e**,**i**) The brighfield image of the HeLa cells treated with Cys (100 μM), PAG (200 μM) and **Na-H**_**2**_ **S-ER** (5 μM); (**j**) The OP Fluorescence image of (**i**,**k**) The merge image of (**i** and **j**,**l**) The TP Fluorescence image of (**i**). The OP mode was excitated at 488 nm and emission collection was from 500–550 nm. The TP mode was excitated at 760 nm and emission collection was from 500–550 nm. Scale bar: 500 μm. Scale bar: 20 μm.

Encouraged by the results of the exogenous H_2_S imaging experiment, we decided to investigate the ability of the fluorescent probe **Na-H**_**2**_ **S-ER** to identify the endogenous H_2_S in the living cells. Researches show that endogenous H_2_S can be generated by the enzymatic reaction of CSE with Cysteine (Cys) in living cells^10^. The cells treated with probe (5 µM) showed non-fluorescence signal (Fig. 5b,d). Under the same imaging parameters, we pre-incubated HeLa cells with Cys (100 µM) and then treated the cells with probe (5 µM), a dramatic fluorescent enhancement was observed by OP and TP modes (Fig. 5f,h). In order to further verify the increased fluorescent signals were mainly generated by the reaction between the probe and the endogenous H_2_S, we carried out a negative control imaging experiment. We conducted the negative control imaging experiment with propargylglycine (PAG) because it decreased the intracellular H_2_S concentration by inhibiting the activity of CSE^44^. As shown in the Fig. 5j,l, with the addition of PAG (200 µM), the fluorescence intensity of the probe decreased significantly whether by OP or TP mode. These data indicated that the probe **Na-H**_**2**_ **S-ER** can monitor endogenous H_2_S in living cells.

### Colocalization imaging experiments

**Figure 6:**
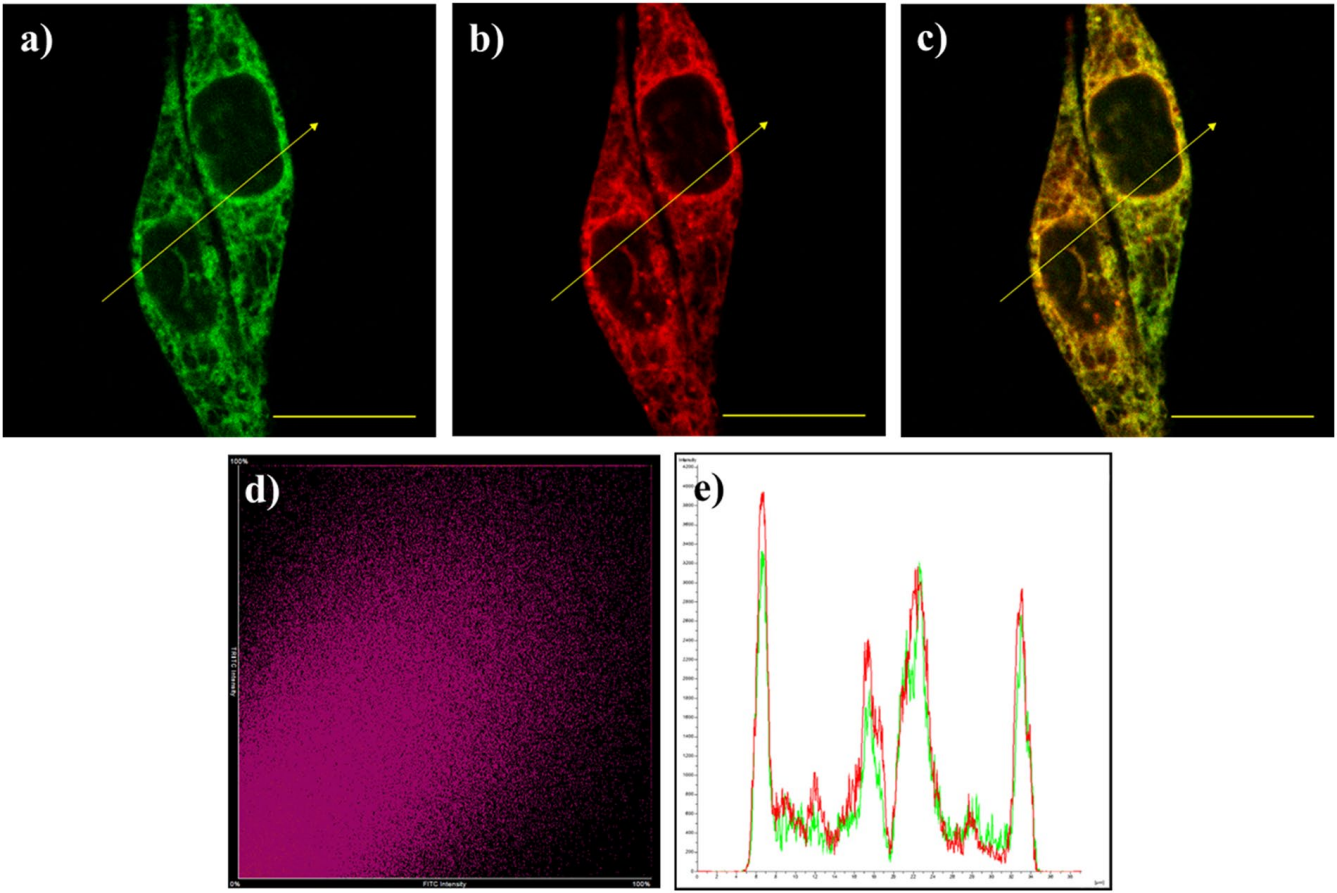
The images of the living HeLa cells co-incubated with the probe **Na-H**_**2**_ **S-ER** (5 μM), Na_2_S (50 μM), and ER-Tracker TM Red. (**a**) The fluorescence image of the green channel; (**b**) The fluorescence image of the red channel; (**c**) The merged image of (**a** and **b**,**d**) Intensity scatter plot of the green and red channels. (**e**) Intensity profile of linear region of interest across in the HeLa cells co-stained with ER-Tracker Red and green channel of **Na-H**_**2**_ **S-ER**. Scale bar: 20 μm.

Encouraged by the above promising results of above imaging experiments, we decided to further verify the sub-cellular distribution of **Na-H**_**2**_ **S-ER** with exogenous H_2_S. Because the methyl sulfonamide group is an effective targeting group for ER, the Hela cells were co-incubated with **Na-H**_**2**_ **S-ER**, Na_2_S and the ER-Tracker TM Red (a commercial available ER Tracker) for the colocalization study (Fig. 6). The HeLa cells showed strong fluorescent signals in the green channel (λ_ex_ = 488 nm; λ_em_ = 500–550 nm) in the presence of Na_2_S. Meanwhile, a significant fluorescence was detected in the red channel (λ_ex_ = 561 nm; λ_em_ = 570–620 nm) because of the gathering of ER Tracker. The merged image showed that the well overlap between the green fluorescence and the red fluorescence (Fig. 6c), and the Pearson’s colocalization coefficient was 0.91 and the Mander’s overlap coefficient was 0.92 (Fig. 6d). The intensity profile of linear regions of interest across HeLa cells in the two channels also varies in close synchrony (Fig. 6e). These data showed that the probe has the prominent property of ER-targeted in the living cells.

### Two-photon fluorescence imaging in living tissues

**Figure 7:**
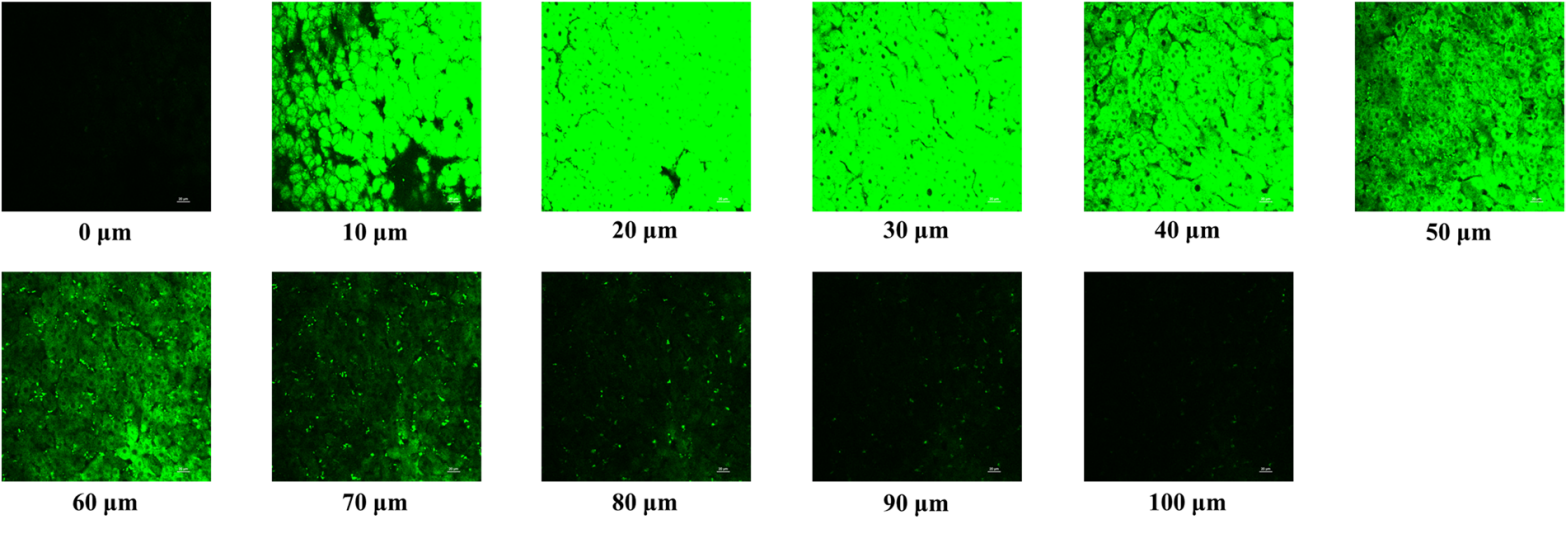
Fluorescence imaging of added H_2_S in living liver tissues. The liver tissue slices pretreated with **Na-H**_**2**_ **S-ER** (30 μM) for 30 min, and then treated with Na_2_S (150 μM) for another 30 min. Excitation was at 760 nm and emission collection was from 500–550 nm. Scale bar: 20 μm.

The beneficial results of the living cell researches led us to further apply **Na-H**_**2**_ **S-ER** to trace the added H_2_S in the living liver tissue. Similar to the cell imaging experiments, the liver tissue slices only incubated with **Na-H**_**2**_ **S-ER** exhibited non-fluorescence (Fig. S13). By contrast, the liver tissue slices treated with the probe and then incubated with added Na_2_S exhibits strong fluorescent signals was observed up to a depth of 100 µm (Fig. 7). Thus, the data showed that the probe is able to detect the addition of H_2_S in liver tissue slides.

### Bio-imaging H_2_S in living zebrafish

**Figure 8:**
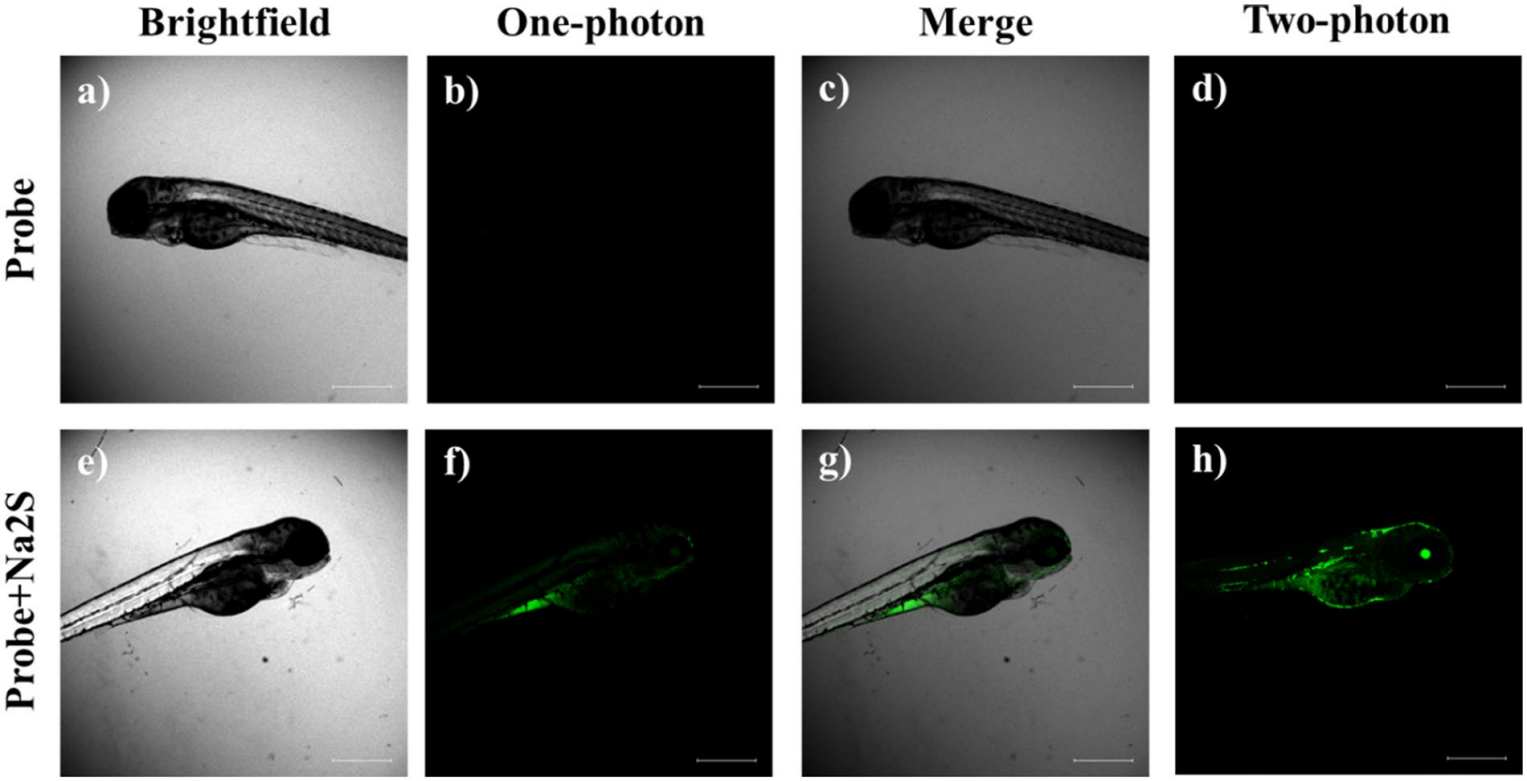
Fluorescence imaging of H_2_S in living zebrafish. (**a**) The brighfield image of the zebrafish treated with **Na-H**_**2**_ **S-ER** (10 μM); (**b**) The OP Fluorescence image of (**a**,**c**) The merge image of (**a** and **b**,**d**) The TP Fluorescence image of (**a**,**e**) The brighfield image of the zebrafish treated with **Na-H**_**2**_ **S-ER** (10 μM) and Na_2_S (50 μM); (**f**) The OP Fluorescence image of (**e**,**g**) The merge image of (**e** and **f**,**h**) The TP Fluorescence image of e. The OP mode was excitated at 488 nm and emission collection was from 500–550 nm. The TP mode was excitated at 760 nm and emission collection was from 500–550 nm. Scale bar: 500 μm.

As a model of good vertebrate animals, zebrafish have been widely used for the study of human genetic diseases and developmental biology^45,46^. However, the study of H_2_S fluorescence imaging using zebrafish as the biological sample is still scarce. Based on the excellent features of **Na-H**_**2**_ **S-ER**, we carried out the fluorescence imaging experiment in the living zebrafish. The zebrafish with feeding **Na-H**_**2**_ **S-ER** were still alive, suggesting that **Na-H**_**2**_ **S-ER** has the potential for biological imaging (Fig. 8a,e). The zebrafish treated only with the probe **Na-H**_**2**_ **S-ER** displayed almost no fluorescence (Fig. 8b,d). However, the zebrafish was fed with both the probe and Na_2_S showed bright fluorescence (Fig. 8f,h), suggesting that **Na-H**_**2**_ **S-ER** is capable of imaging added H_2_S in living zebrafish.

## Conclusions

We had engineered the first ER-targeted two-photon H_2_S fluorescent probe **Na-H**_**2**_ **S-ER**. The fluorescence probe contains the fluorescent platform 1,8-naphthalimide and the responding site azide group. When introduced of the H_2_S to **Na-H**_**2**_ **S-ER**, a large fluorescent emission signal at 545 nm was observed because of the azide group reduced by H_2_S to the amine moieties. The probe **Na-H**_**2**_ **S-ER** possessed potential biological imaging value because it has many excellent characteristics, including large fluorescent enhancement signals, high sensitivity and selectivity, low cytotoxicity, and working appropriately at the physiological pH. Remarkably, this probe could be applied to monitor the dynamic changes of H_2_S in ER of the living cells because it possesses the excellent ER-targeted feature. Importantly, the probe has successfully carried out the tissue and zebrafish imaging experiments. We believe that **Na-H**_**2**_ **S-ER** as a powerful molecular tool for monitoring H_2_S in biological samples.

## Methods

### Synthesis of the probe Na-H_2_S-ER

The synthesis of compound **2**, **4** was referred to the previous literature^41^. 472 mg compound **4** (1 mmol) and 325 mg sodium azide (5 mmol) were added to 5 mL of anhydrous DMF. The reaction mixture was heated at 50 °C overnight under the nitrogen atmosphere. Then 50 mL H_2_O was added to the reaction system, then extracted with dichloromethane (20 mL × 3). The organic phase was washed with H_2_O, dried with MgSO_4_. The crude product was purified by flash chromatography to afford a yellow solid **Na-H**_**2**_ **S-ER** 249 mg with a yield of 57%. ^1^H NMR (400 MHz, DMSO-*d*_6_) δ 8.49 (d, *J* = 7.2 Hz, 1 H), 8.42 (m, 2 H), 7.86 (t, *J* = 8.0 Hz, 1 H), 7.76 (m, 2 H), 7.58 (d, *J* = 8.0 Hz, 2 H), 7.23d, *J* = 8.0 Hz, 2 H), 4.09 (t, *J* = 8.0 Hz, 2 H), 3.08 (q, *J* = 5.6 Hz, 2 H), 2.26 (s, 3 H)^13^; C NMR (100 MHz, DMSO-*d*_6_) δ 163.75, 163.30, 143.23, 142.89, 138.14, 131.96, 131.88, 129.93, 128.68, 128.75, 127.71, 126.80, 123.96, 122.70, 118.72, 116.36, 21.32; HR-MS calculated for C_21_H_17_N_5_O_4_S [M + H]^+^ m/z 436.1074, found 436.1070.

### Synthesis of the compound Na-ER

A mixture of compound **Na-H**_**2**_ **S-ER** 87 mg (0.2 mmol) was dissolved in 95% EtOH (1 mL), and then 80 mg Na_2_S·9H_2_O (1 mmol) was added. The suspension was stirred at room temperature for 1 h. Subsequently, the mixture was concentrated under vacuum, and the resulting residue was purified by silica gel column chromatography (CH_2_Cl_2_/MeOH as the eluent) to afford 67 mg orange solid of the compound **Na-ER** with a yield of 83%. ^1^H NMR (400 MHz, DMSO-*d*_6_) δ 8.61 (d, *J* = 8.0 Hz, 1 H), 8.38 (d, *J* = 6.8 Hz, 1 H), 8.15 (d, *J* = 8.0 Hz, 1 H), 7.71 (t, *J* = 6.0 Hz, 1 H), 7.64 (t, *J* = 8.0 Hz, 1 H), 7.59 (d, *J* = 8.0 Hz, 2 H), 7.46 (s, 2 H), 7.23 (d, *J* = 8.0 Hz, 2 H), 6.83 (d, *J* = 8.0 Hz, 1 H), 4.07 (t, *J* = 6.4 Hz, 2 H), 3.02 (q, *J* = 6.4 Hz, 2 H), 2.27 (s, 3 H); ^13^C NMR (100 MHz, DMSO-*d*_6_) δ 164.32, 163.38, 153.17, 142.86, 138.06, 134.36, 131.38, 130.26, 129.89, 129.72, 126.84, 124.35, 122.25, 119.81, 108.57, 108.04, 40.79, 39.20, 21.33; HR-MS calculated for C_21_H_19_N_3_O_4_S [M + H]^+^ m/z 410.1169, found 410.1162.

### Spectral analysis

Unless otherwise noted, all the measurements were made according to the following procedure. The concentration of the probe stock solution was 1.0 mM in DMSO, and the analytes stock solutions were prepared in the ultrapure water at the appropriate concentration. In 5 mL volumetric flask, the test solution was prepared by placing 25 μL probe stock solution and 225 μL DMSO, requisite amount of analyte stock solution, then adjusted the final volume to 5 mL with PBS buffer. The spectrum tests were recorded with a 1 cm standard quartz cell at room temperature. The absorption spectra were obtained on a Shimadzu UV-2700 Power spectrometer. The photoluminescent spectra were recorded with a HITACHI F4600 fluorescence spectrophotometer. The excitation wavelength was 440 nm, the excitation slit widths were 5 nm, and the emission slit widths were 5 nm.

### Cytotoxicity assay

The HeLa cells were seeded up to appropriate density in a 96-well plate. Then the cells were incubated with a series of concentrations of **Na-H**_**2**_ **S-ER** (0–20 μM) at 37 °C. After 24 h, the cells were with PBS buffer, then added MTT (5 mg/mL, 10 μL) and further incubated for 4 h. After removed the culture medium, DMSO (100 μL) was added into the dishes to dissolve the formazan crystal product. Then the absorbance of the solution was measured at 570 nm by the microplate reader.

$${\rm{The}}\,{\rm{cell}}\,{\rm{viability}}\,( \% )=({{\rm{OD}}}_{{\rm{sample}}}-{{\rm{OD}}}_{{\rm{blank}}})/({{\rm{OD}}}_{{\rm{control}}}-{{\rm{OD}}}_{{\rm{blank}}})\times 100 \% .$$

OD_sample_ denotes the cells incubated with various concentrations of the probe, OD_control_ denotes the cells without the probe, OD_blank_ denotes the wells containing only the culture medium.

### Bio-imaging of exogenous H_2_S in living HeLa cells

The HeLa cells were seeded up to appropriate density into a 35 mm glass-bottom culture dishes (Nest). Then the cells further incubated with **Na-H**_**2**_ **S-ER** (5 μM) for another 20 min at 37 °C. Then the cells were washed with PBS buffer (pH = 7.4) three times, and the cells incubated with Na_2_S (50 μM) for more 30 min. Finally, the cells were washed three times with PBS buffer. The imaging experiments were recorded through a Nikon A1MP confocal microscopy inverted fluorescence microscopy equipped with a cooled CCD camera. The OP fluorescence emission was collected at 500–550 nm upon excitation at 488 nm with a femtosecond pulse, and the TP fluorescence emission was collected at 500–550 nm upon excitation at 760 nm with a femtosecond pulse.

### Bio-imaging of endogenous H_2_S in living HeLa cells

For the endogenous H_2_S group, the cells further incubated with Cys (100 μM) for another 30 min at 37 °C, then washed with PBS three times and further incubated with **Na-H**_**2**_ **S-ER** (5 μM) for another 20 min. For negative control group, the cells incubated with Cys (100 μM) and PAG (200 μM) for 30 min, then further incubated with **Na-H**_**2**_ **S-ER** (5 μM) for another 20 min. Finally, the cells were washed three times with PBS buffer. Then the imaging experiments were carried out.

### Bio-imaging of added H_2_S in living tissue

4 weeks old Kunming mice were purchased from Shandong University Laboratory Animal Centre (Shandong, China). Unless otherwise noted, all procedures for this study were approved by the Animal Ethical Experimentation Committee of Shandong University according to the requirements of the National Act on the use of experimental animals (China). The mice were killed by cervical vertebra dislocation, the liver tissues were cut into about 500 μm in size. For the control group, the liver tissue slices treated with **Na-H**_**2**_ **S-ER** (30 μM) for 30 min. For the experimental group, the liver tissue slices pretreated with **Na-H**_**2**_ **S-ER** (30 μM) for 30 min, and then treated with Na_2_S (150 μM) for another 30 min. Then the imaging experiments were carried out.

### Bio-imaging of exogenous H_2_S in living zebrafish

Wild type zebrafish were purchased from the *Nanjing EzeRinka Biotechnology Co., Ltd*. All procedures for this study were approved by the Animal Ethical Experimentation Committee of Shandong University according to the requirements of the National Act on the use of experimental animals (China). For the control group, the zebrafish were fed with **Na-H**_**2**_ **S-ER** (10 μM) for 30 min. For the experimental group, the zebrafish were fed with **Na-H**_**2**_ **S-ER** (10 μM) for 30 min, and then treated with Na_2_S (50 μM) for another 30 min. Then the imaging experiments were carried out.

## Electronic supplementary material

Supplementary Information
