# Supplementary material for: A turn-on endoplasmic reticulum-targeted two-photon fluorescent probe for hydrogen sulfide and bio-imaging applications in living cells, tissues, and zebrafish
Source: Sci Rep. 2017 Oct 11;7:12944. doi: 10.1038/s41598-017-13325-z (PMC5636802; doi:10.1038/s41598-017-13325-z)
Supplement: Supplementary file 1 — Supplementary Information [file 41598_2017_13325_MOESM1_ESM.pdf]

Supporting Information for

A turn-on endoplasmic reticulum-targeted two-photon fluorescent probe for hydrogen sulfide and bio-imaging applications in living cells, tissues, and zebrafishes

*Yonghe Tang, An Xu, Yanyan Ma, Gaoping Xu, Shiying Gao, and Weiying Lin**

Institute of Fluorescent Probes for Biological Imaging, School of Chemistry and Chemical Engineering, School of Materials Science and Engineering, University of Jinan, Jinan, Shandong 250022, P.R. Email: weiyinglin2013@163.com

*Correspondence to: Weiying Lin, Institute of Fluorescent Probes for Biological Imaging, School of Chemistry and Chemical Engineering, School of Materials Science and Engineering, University of Jinan, Jinan, Shandong 250022, P.R. China. Email: weiyinglin2013@163.com.

Table of Content

page

Figures. S1-S2. .... s3

Figure. S3. .... s4

Figures. S4-S5. .... s5

Figures. S6-S7. .... s6

Figure. S8. .... s7

Figures. S9-S10. .... s8

Figures. S11-S12. .... s9

Figures. S13-S14 .... s10

Figures. S15-S16. .... s11

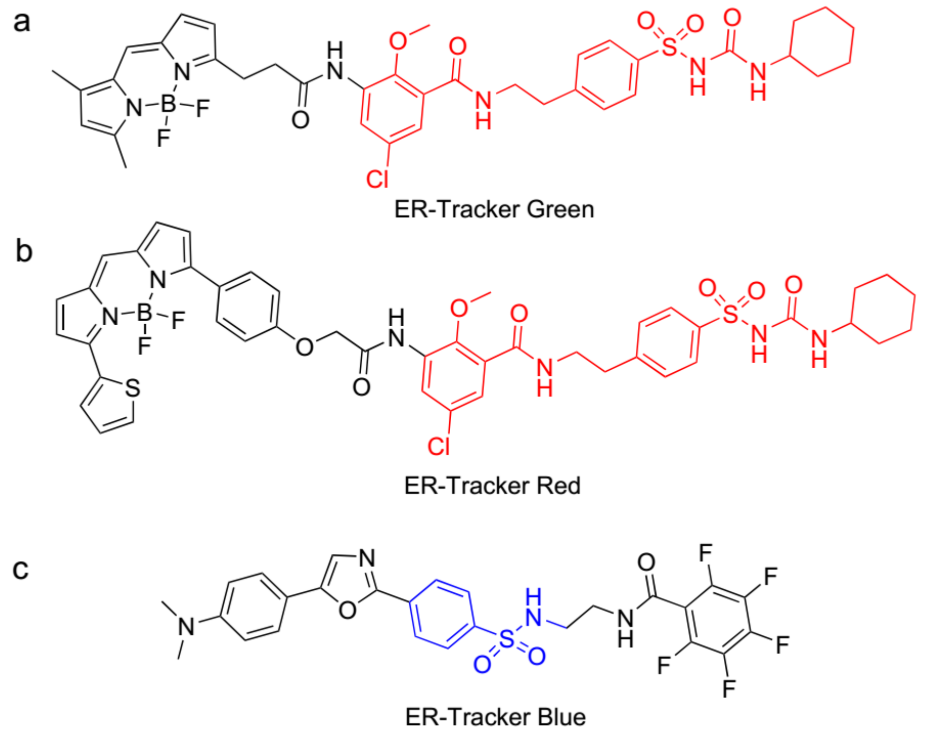

Figure. S1. The Structure of ER-tracker Green (a), ER-tracker Red (b), and ER-tracker Blue (c).

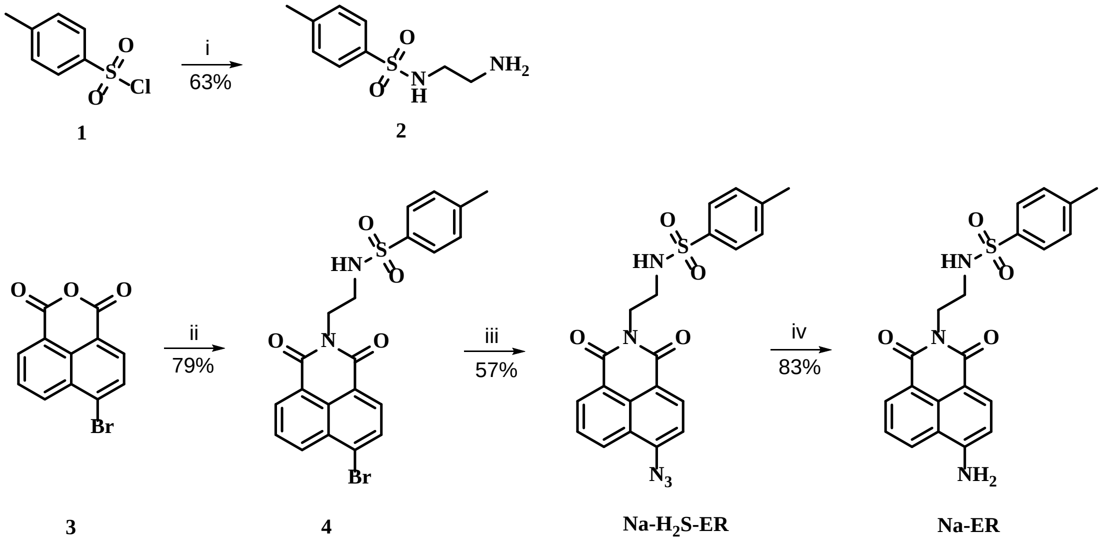

Figure. S2. Synthesis of the fluorescent formaldehyde probe **Na-H_2S-ER** and **Na-ER.** Reagents and conditions: (i) Ethylenediamine, CH_2Cl_2, room temperature, 12h; (ii) Compound **2**, EtOH, reflux, 3h; (iii) Sodium azide, DMF, 50℃, 4h. (iv) Na_2S, 95 % EtOH, room temperature, 1 h.

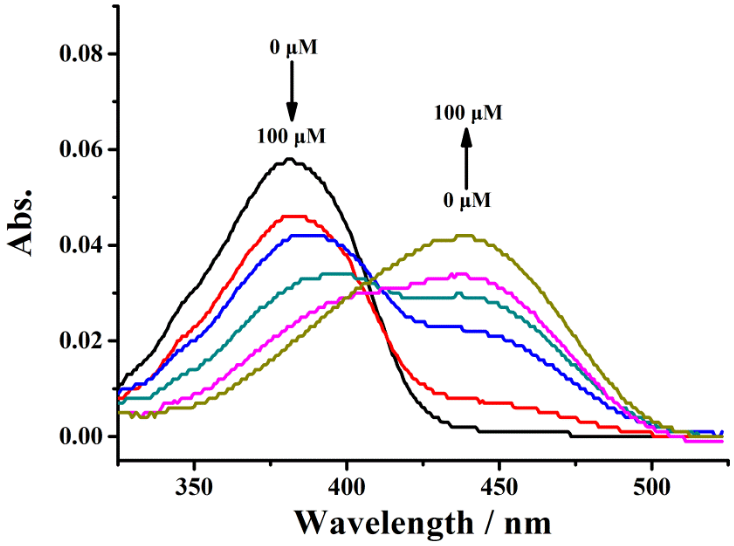

Figure. S3. The absorption spectral changes of **Na-H_2S-ER** (5 μM) upon addition of increasing concentrations of Na_2S (0-100 μM) in PBS buffer (pH 7.4, 5 % DMSO).

Detection limit: The detection limit was determined from the fluorescence titration data based on a reported method [S1]. The probe **Na-H_2S-ER** (5 μM) was titrated with Na_2S (0-150 μM) for 30 min. The fluorescent intensity data at 545 nm were normalized between the minimum intensity and the maximum intensity. A linear regression curve was then fitted to the normalized fluorescent intensity data and the point at which this line crossed the axis was considered as the detection limit (7.77×10^{-6} M).

[S1] Shortreed, M., et al. Fluorescent Fiber-Optic Calcium Sensor for Physiological Measurements. *Anal. Chem.* **68**, 1414-1418 (1996).

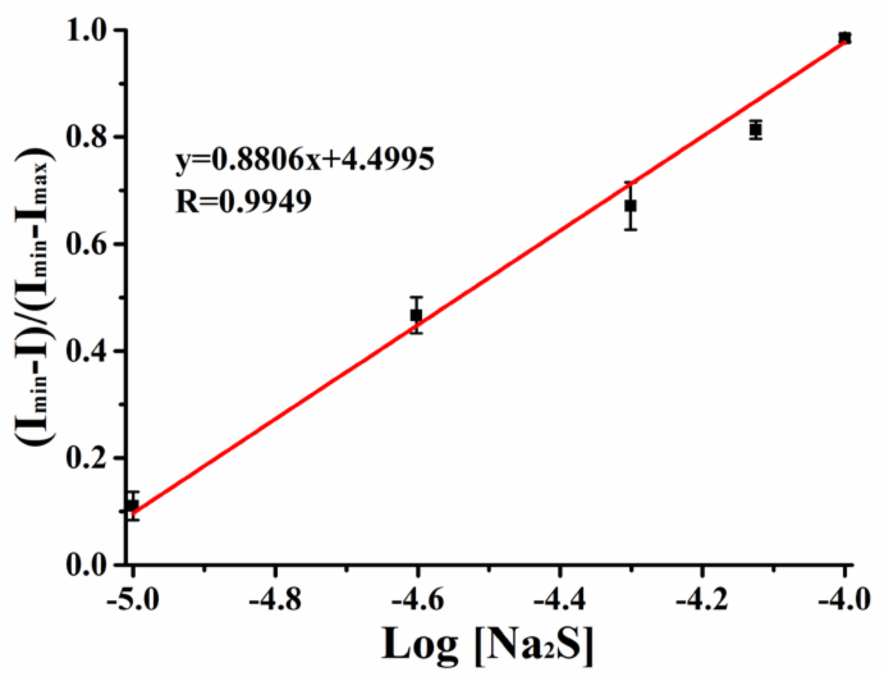

Figure. S4. Normalized response of the fluorescence signal by changing the concentration of Na_2S.

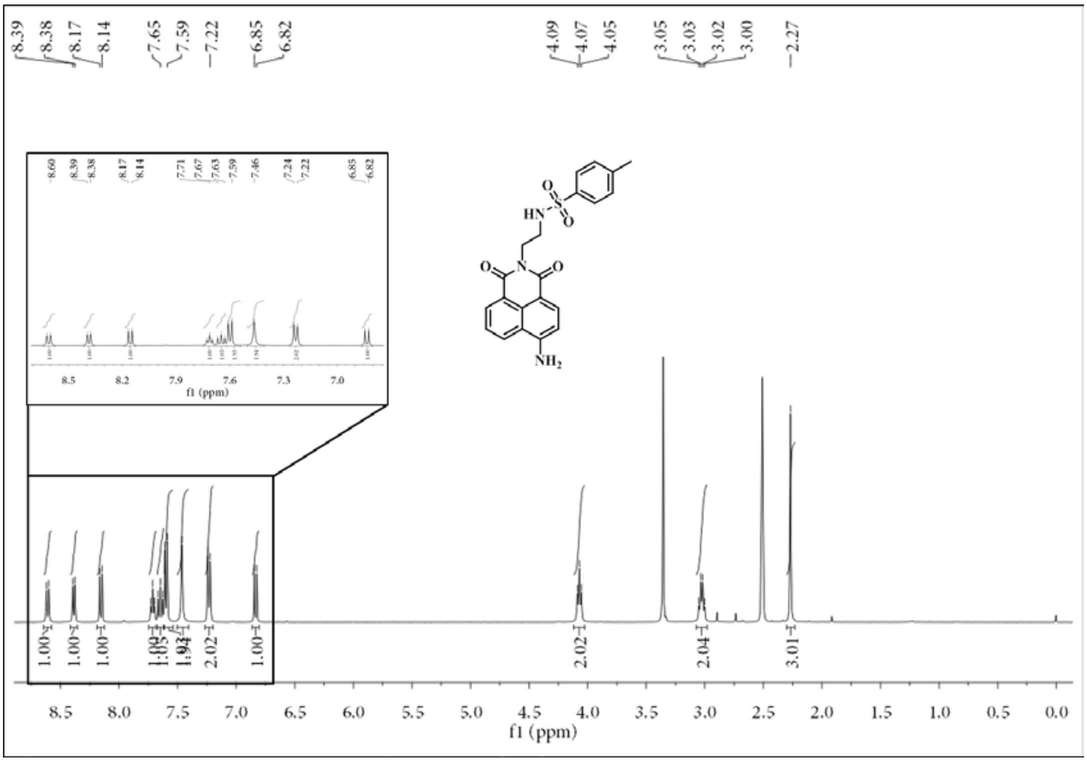

Figure. S5. ^{1}H NMR spectrum of the compound **Na-ER**.

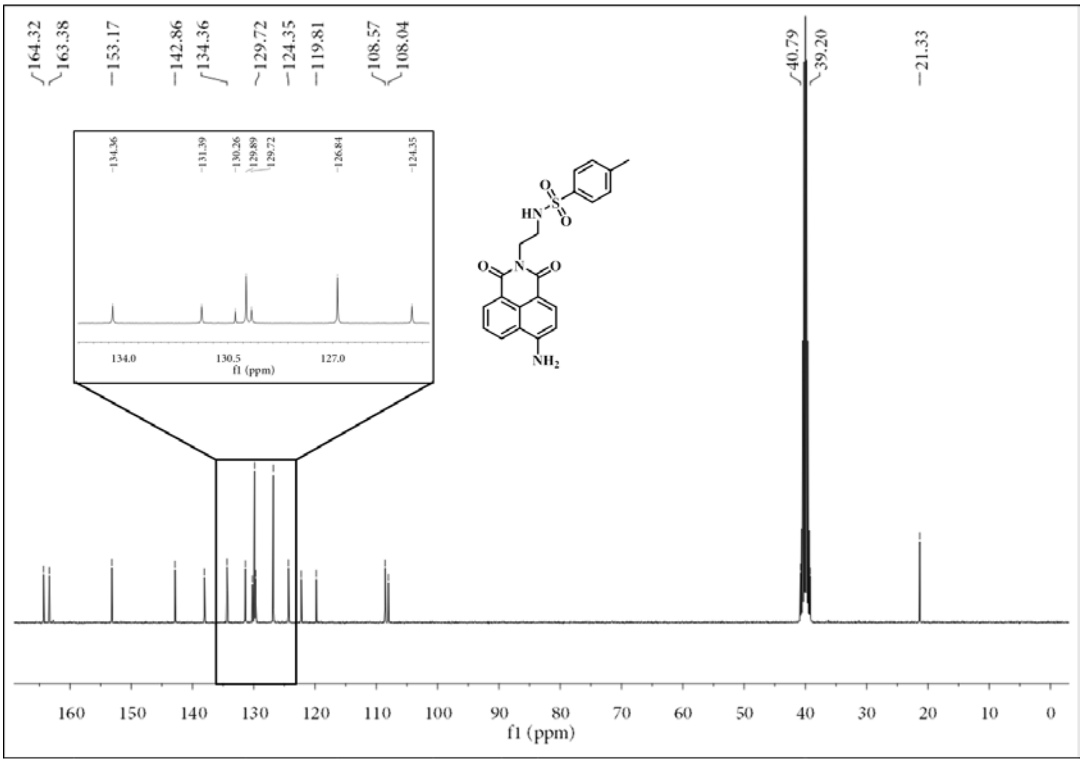

Figure. S6. ^{13}C NMR spectrum of the compound **Na-ER**.

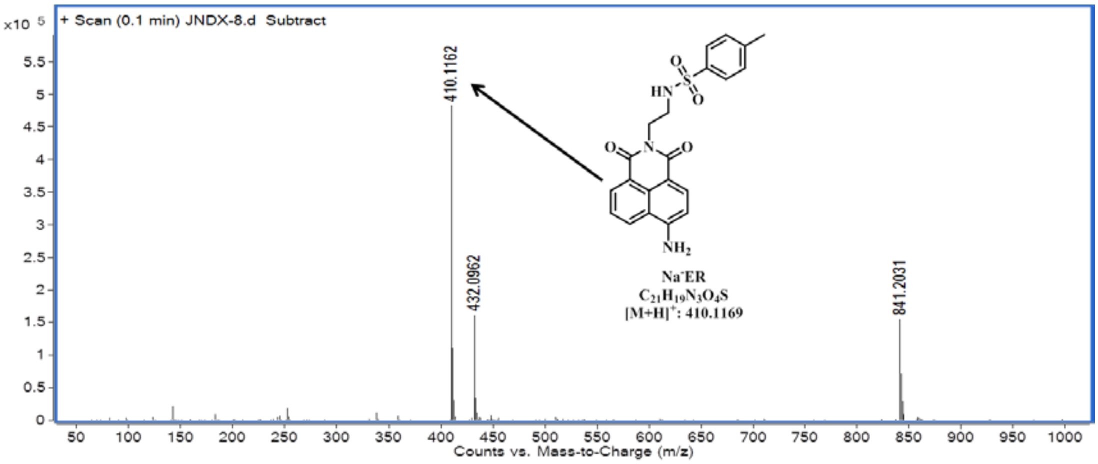

Figure. S7. HR-MS spectrum of the compound **Na-ER**.

Kinetic studies: The rate constant was determined from the fluorescence titration data based on a reported method [S2]. The reaction of the probe **Na-H_2S-ER** (5 μM) with Na_2S at varied concentrations in PBS (10 mM, pH 7.4, 5 % DMSO) was monitored using the fluorescence intensity at 545 nm. The reaction was carried out at room temperature. The *pseudo*-first-order rate constant for the reaction was determined by fitting the fluorescence intensities of the samples to the *pseudo*-first-order equation:

$$Ln[(F_{max} - F_t) / F_{max}] = - k' t. \qquad (2)$$

Where F_t and F_{max} are the fluorescence intensities at 545 nm at time t and the maximum value obtained after the reaction was complete. k' is the *pseudo*-first order rate constant. The *pseudo*-first-order plots for the reaction of **Na-H_2S-ER** with 20 equiv. of Na_2S is shown in Figure. S7, The negative slope of the line provides the *pseudo*-first-order rate constant for Na_2S.

[S2] Dale, T. J. & Rebek, J. Fluorescent Sensors for Organophosphorus Nerve Agent Mimics. *J. Am. Chem. Soc.* **128**, 4500-4501 (2006).

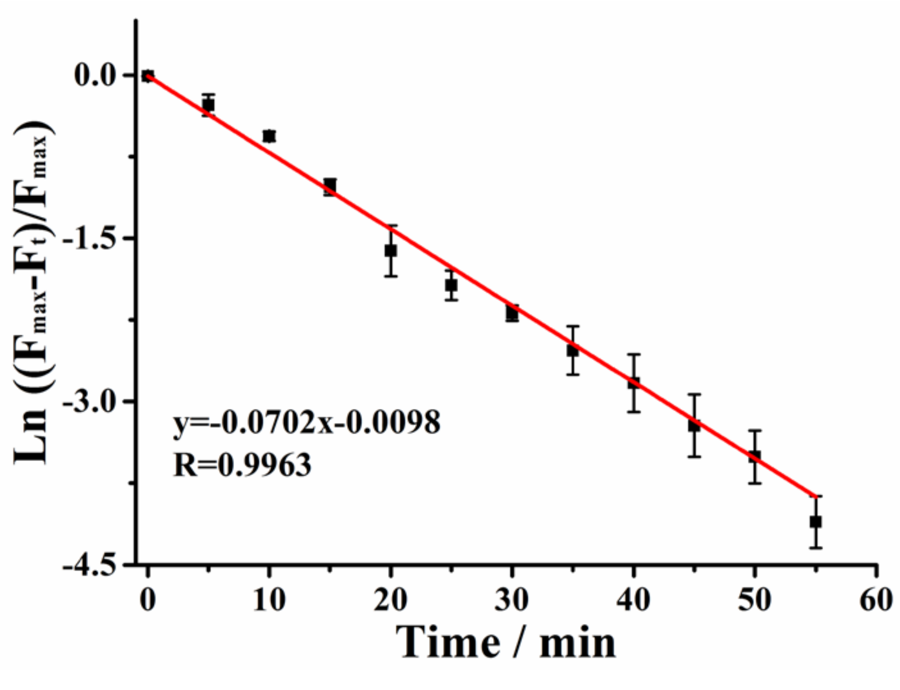

Figure. S8. *Pseudo*-first-order kinetic plot of the reaction of **Na-H_2S-ER** (5 μM) with Na_2S (20 equiv.) in PBS (10 mM, pH 7.4, 5% DMSO). Slope = 0.0702 min^{-1}.

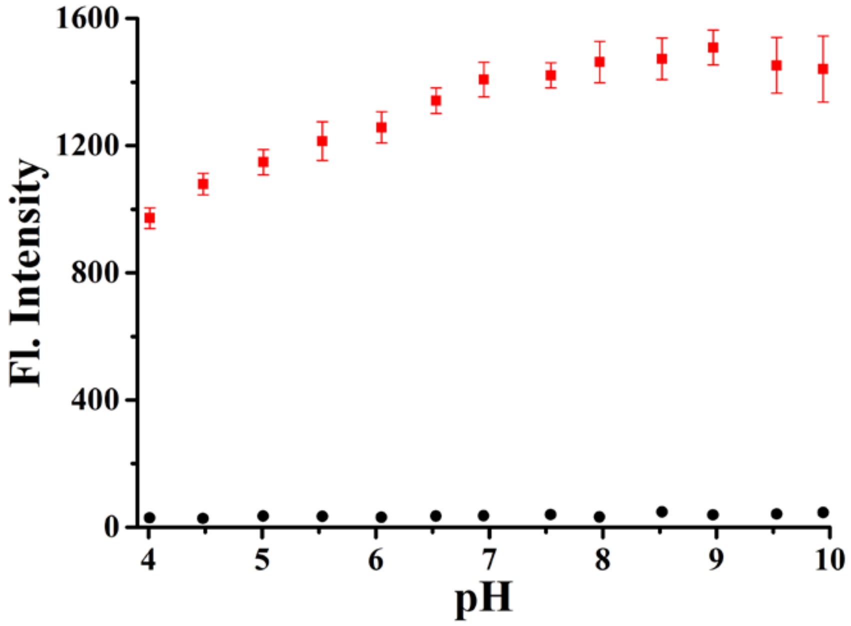

Figure. S9. Fluorescence intensity changes of the probe **Na-H_2S-ER** (5 μM) at different pH values in the absence (■) or presence (●) of Na_2S (100 μM).

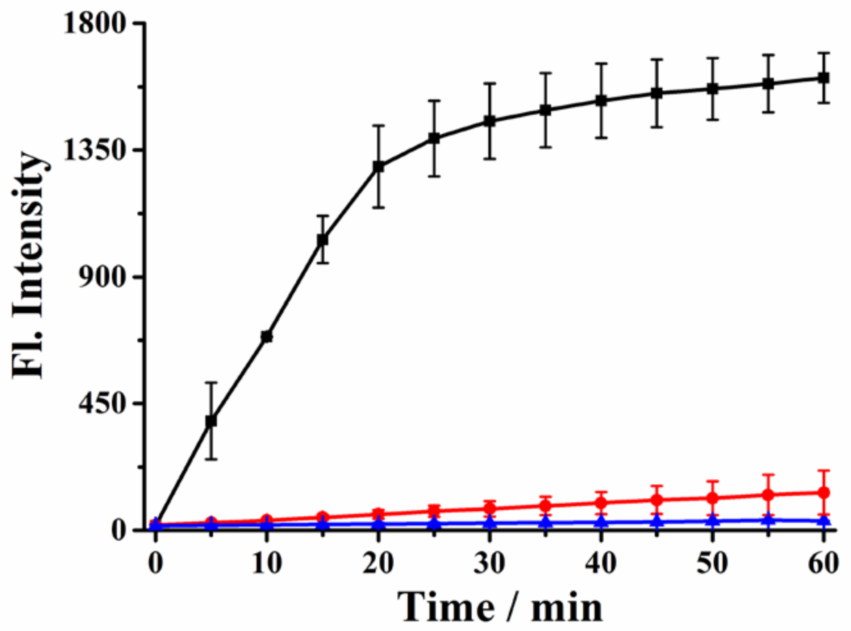

Figure. S10. Photostability profiles of the free probe **Na-H_2S-ER** (5.0 μM) in the absence [▲] or presence of UV-irradiated [●] (365 nm). And the red circle[■] stand for the probe **Na-H_2S-ER** (5.0 μM) treated with Na_2S (100 μM). The fluorescence intensities at 545 nm were continuously monitored at time intervals in PBS (10 mM, pH 7.4, 5 % DMSO). Time points represent 0, 5, 10, 15, 20, 25, 30, 35, 40, 45, 50, 55, and 60 min.

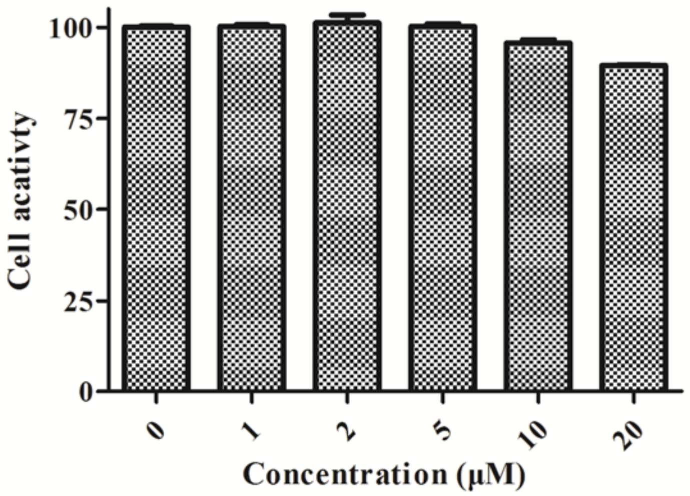

Figure. S11. Effects of the probe **Na-H$_2$S-ER** with varied concentrations (0-20 μM) on the viability of the Hela cells. The probe with varied concentrations was incubated with the cells for 24 h. The viability of the cells in the absence of the probe is defined as 100 %, and the data are the mean standard deviation of five separate measurements.

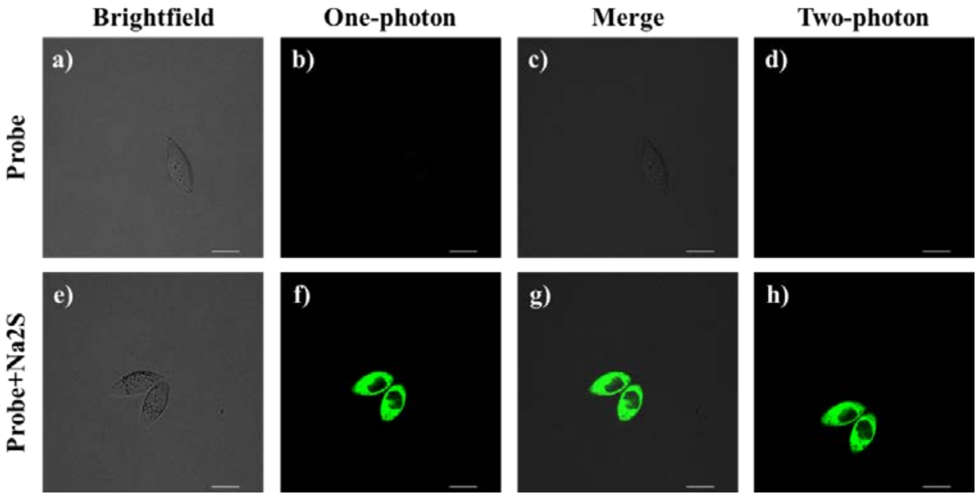

Figure. S12. Fluorescence imaging of the added H_2S in the HeLa cells. a) The brighfield image of the HeLa cells treated with **Na-H$_2$S-ER** (5 μM); b) The OP Fluorescence image of a; c) The merge image of a and b; d) The TP Fluorescence image of a; e) The brighfield image of the HeLa cells treated with **Na-H$_2$S-ER** (5 μM) for 30 min, and then treated with Na_2S (50 μM) for another 30 min; f) The OP Fluorescence image of e; g) The merge image of e and f; h) The TP Fluorescence image of e. Excitation was at 488 nm and emission collection was from 500-550 nm. Scale bar: 20 μm.

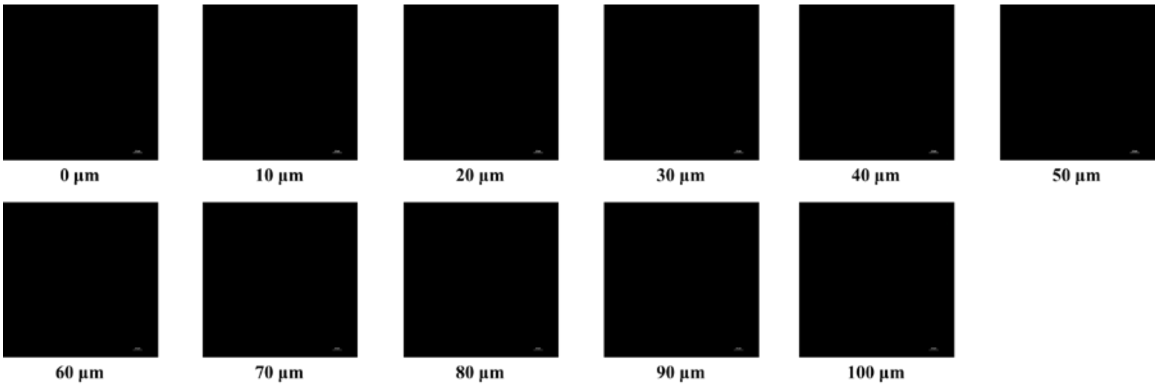

Figure. S13. Fluorescence imaging of **Na-H_2S-ER** in living liver tissues. The liver tissue slices pretreated with **Na-H_2S-ER** (30 µM) for 30 min. Excitation was at 760 nm and emission collection was from 500-550 nm. Scale bar: 20 µm.

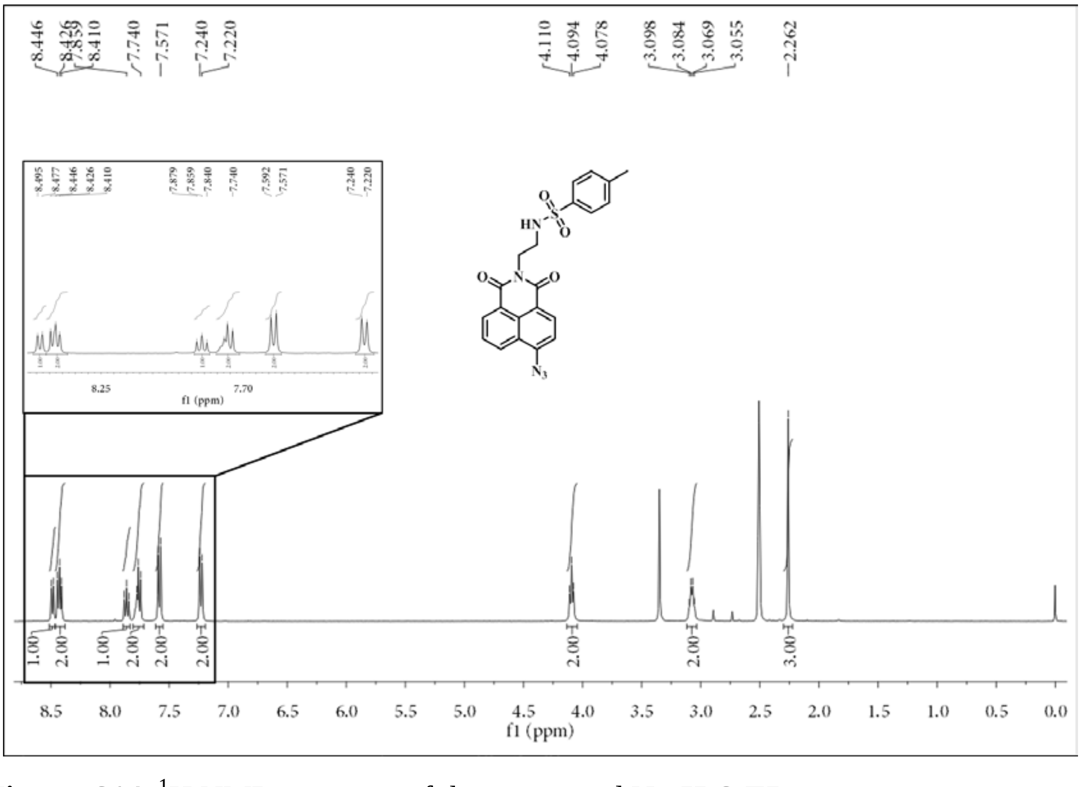

Figure. S14. ^{1}H NMR spectrum of the compound **Na-H_2S-ER**.

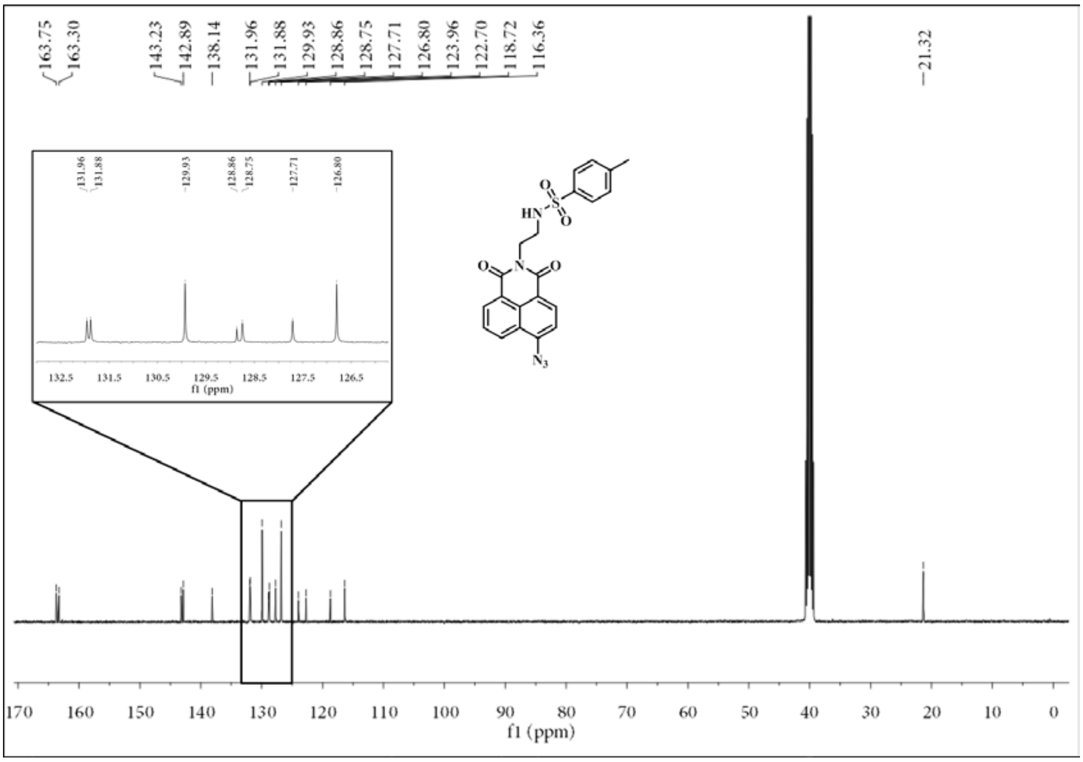

Figure. S15. ^{13}C NMR spectrum of the compound **Na-H$_2$S-ER**.

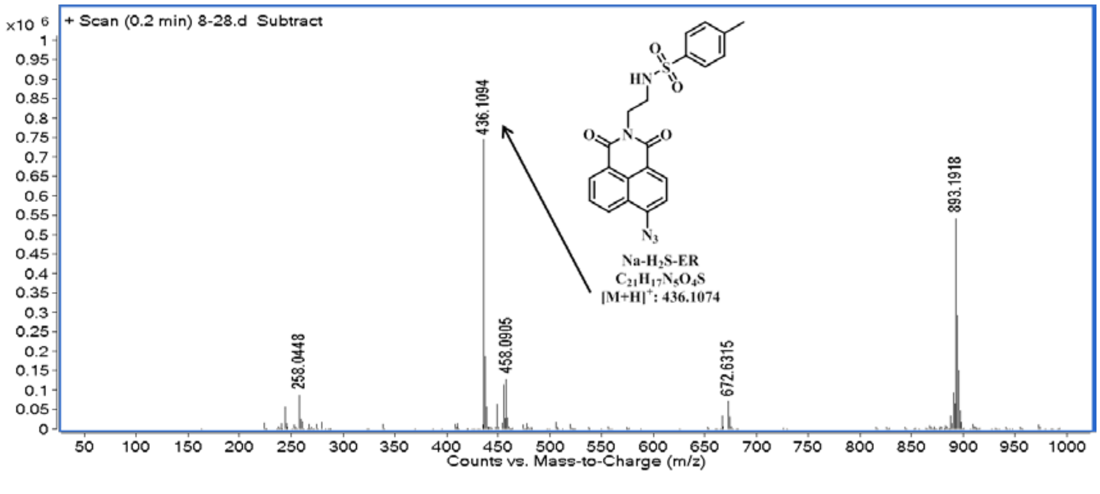

Figure. S16. HR-MS spectrum of the compound **Na-H$_2$S-ER**.
